# New targets for resolution of airway remodeling in obstructive lung diseases

**DOI:** 10.12688/f1000research.14581.1

**Published:** 2018-05-30

**Authors:** Ajay P. Nayak, Deepak A. Deshpande, Raymond B. Penn

**Affiliations:** 1 Center for Translational Medicine, Department of Medicine, Thomas Jefferson University, Philadelphia, USA

**Keywords:** airway remodeling, asthma, GPCR, smooth muscle

## Abstract

Airway remodeling (AR) is a progressive pathological feature of the obstructive lung diseases, including asthma and chronic obstructive pulmonary disease (COPD). The pathology manifests itself in the form of significant, progressive, and (to date) seemingly irreversible changes to distinct respiratory structural compartments. Consequently, AR correlates with disease severity and the gradual decline in pulmonary function associated with asthma and COPD. Although current asthma/COPD drugs manage airway contraction and inflammation, none of these effectively prevent or reverse features of AR. In this review, we provide a brief overview of the features and putative mechanisms affecting AR. We further discuss recently proposed strategies with promise for deterring or treating AR.

## Airway remodeling in obstructive lung diseases

Airway remodeling (AR) can be defined as a progressive pathological reorganization of the cellular and molecular constitution of the airway wall. While the onset and rate of progression of structural changes in the airways have been subjects of immense debate, AR has been associated with each of the asthmatic phenotypes ^1^. Furthermore, the gradual deleterious transformation in lungs can affect airways of all sizes along the bronchial tree. Although the strategies for reversing airway contraction and mitigating airway inflammation have been mainstays of asthma therapy, AR has been clinically intractable. Consequently, a pressing need exists for defining the fundamental pathways contributing to AR pathology and for empowering both basic and clinical research to address this problem. For a comprehensive understanding of the conceptual and practical challenges in AR research, readers are encouraged to review the official research statement of the American Thoracic Society ^2^. In this current report, we provide an overview of the limitations of currently approved anti-asthma/chronic obstructive pulmonary disease (COPD) drugs in addressing AR and further describe the therapeutic potential of recently proposed approaches for targeting AR.

AR was first described in 1922 in patients whose death was attributed to asthma. Necropsy specimens from these patients revealed extensive bronchial mucus plugs and thickening of the airway wall ^3^. Numerous subsequent clinical investigations have revealed that AR encompasses broad structural changes in the airway that includes thickening of the airway wall, airway smooth muscle (ASM) hyperplasia and hypertrophy, edema, subepithelial fibrosis, increased extracellular matrix (ECM) deposition, immune cell and fibroblast accumulation, angiogenesis, altered matrix composition, goblet cell metaplasia, and mucus hypersecretion ^2^. A consensus has emerged that multiple cell types (including epithelium, ASM, fibroblasts, and immune cells) contribute to the development of AR in asthma and COPD ^4, 5^ ( Table 1).

**Table 1.** Contribution by distinct cell types to the overall pathology of airway remodeling in obstructive lung diseases.

Lung cell type	Contribution to the pathophysiology of airway remodeling
Epithelial cells	Epithelial shedding
	Mucus secretion
	Subepithelial fibrosis
	Goblet cell hyperplasia
	Stimulating airway smooth muscle (ASM) proliferation through release of growth factors
	Recruitment of pro-inflammatory cells
	Promoting extracellular matrix (ECM) deposition
	Promoting angiogenesis
ASM cells	Increased ASM mass
	ASM migration and invasion of the epithelium
	Adoption of synthetic phenotype (for example, secretion of transforming growth factor-beta, chemokines, and ECM components)
	Interaction with immune cells through cell adhesion molecules
Fibroblasts	Differentiation into myofibroblasts and secretion of ECM components
	Accumulation in subepithelial regions

The role of the airway epithelium in triggering initial responses and sustaining architectural changes in asthmatic lungs is evident ^6, 7^. In asthma, repetitive damage to the epithelium from exposure to noxious environmental agents and immune modulators promotes shedding of the epithelium. Consequently, the underlying epithelial-mesenchymal trophic unit may be persistently active and in a reparative state, thus promoting chronic and progressive remodeling of the airway ^8^. Remodeling manifests in the form of thickening of the epithelial layer, loss of cilia, compromised barrier function, mucus hypersecretion, and ECM remodeling of the subepithelial space ^6, 7, 9– 17^. Moreover, the number of mucus-secreting goblet cells also increases in asthmatics ^18, 19^. These features collectively contribute to anatomical changes that cause airway narrowing, increased fixed resistance, and mucus plugging of the bronchial lumen.

Physiological ASM function is crucial for maintaining adequate airflow. Changes in both ASM responsiveness and morphology occur with asthma, which affects airway resistance and airflow. A critical feature of AR is an increase in ASM mass that contributes significantly to asthma pathology ^20, 21^. Furthermore, the increased ASM mass and increased airway wall thickness reduce airway lumen area, resulting in increased dynamic and fixed resistance ^21– 26^. Asthmatic ASM can also acquire a synthetic phenotype, which is characterized by increased secretion of ECM, cytokines, and growth factors. Clinical outcomes associated with bronchial thermoplasty intervention (application of controlled radiofrequency energy to the airway wall) suggest that reducing ASM area is sufficient to improve outcomes in asthmatics ^27^.

Fibroblasts can contribute to AR through increased secretion of ECM ^28, 29^. Beyond contributing to increased airway wall thickness, ECM components can modulate cellular proliferation and migration. However, the role of fibroblast and ECM components in AR in the context of obstructive lung diseases is not fully understood.

The structural changes may contribute toward a gradual decline in lung function and potentially in loss of pulmonary elasticity, leading to hyperinflation and air trapping in lungs. Moreover, remodeling reduces effectiveness of bronchodilatory treatments ^5, 30– 32^. A correlation between AR and disease severity has been established, but the clinical consequences of AR are yet to be fully understood ^33– 38^. This lack of knowledge also impacts drug discovery efforts. In the subsequent sections, we review the efficacy of current therapeutics in blunting or reversing AR and discuss novel therapeutic approaches to regulate progression of AR.

## Overview of current therapeutics and their limitations

Current management of asthma focuses on reversing ASM contraction and mitigating airway inflammation. None of these approaches directly addresses the progressive pathology that causes remodeling in the lung ( Table 2).

**Table 2.** Current therapeutic targets for asthma management and their effect on airway remodeling.

Class of therapeutic drugs	Target	Effect on airway remodeling
β-agonists	β _2_-AR (beta 2 adrenergic receptor)	Limited effect on airway remodeling ^5^. Combination therapy with inhaled corticosteroid limits angiogenesis and fibroblast proliferation ^55, 56, 71^.
Inhaled corticosteroids	Glucocorticoid receptor	Combination therapy with β-agonists limits angiogenesis ^71^. Mixed anti- proliferative actions on airway smooth muscle cells and human fibroblasts ^55, 56^. Reduced mucin secretion and limited extracellular matrix deposition ^61– 64^.
Anti-leukotrienes	CysLTR (cysteinyl leukotriene receptor)	Moderate effect on airway smooth muscle mass, goblet cell metaplasia, and subepithelial collagen deposition ^45, 46, 62^. Decreased accumulation of fibroblasts in lungs ^47^.

As noted earlier, bronchial thermoplasty has been shown to reduce ASM mass in conducting airways of some, but not all, severe asthmatics undergoing the procedure ^27, 39, 40^. This procedure has been shown to significantly reduce collagen deposition in the basement membrane. Although bronchial thermoplasty has been shown to improve quality of life for severe asthmatics in the short term, the cost of the procedure, post-procedure exacerbations, and questions regarding long-term efficacy have limited its application ^41^.

Among pharmacological options, β-agonists are the drug of choice for evoking bronchorelaxation in attempting to reverse an acute asthma attack or for providing bronchoprotection when used in combination with an inhaled corticosteroid as a maintenance therapy. However, there is no compelling evidence that β-agonists deter or reverse AR ^5^. Signaling through cysteinyl leukotriene receptors (CysLTRs) and muscarinic acetylcholine receptors (mAChRs) has been established to promote outcomes that contribute to AR ^42– 44^. Antagonists of both receptors have shown some utility in preventing AR. Treatment with the CysLTR antagonist montelukast reversed ovalbumin-induced AR by decreasing goblet cell metaplasia, ASM mass, and subepithelial collagen deposition ^45, 46^. In a cohort of mild asthmatics, montelukast treatment showed reduced accumulation of myofibroblasts in the airway wall, suggesting some potential to mitigate AR ^47^. Similarly, the long-acting mAChR antagonist, tiotropium, has demonstrated a robust ability in preventing AR in rodent (guinea pig and mouse) models of ovalbumin-induced asthma and lipopolysaccharide-induced COPD ^48– 52^. Overall, although some evidence suggests that mAChR and CysLTR antagonists may have utility in deterring AR, additional studies in humans are necessary to establish the true effectiveness of these drugs in preventing or reversing AR ^5^.

Persistent asthma is commonly treated with inhaled corticosteroids either as a monotherapy or in combination with a β-agonist or mAChR antagonist. In epithelial cells, corticosteroids limit the inflammatory response and induce apoptosis ^53, 54^. *In vitro*, multiple corticosteroids have been shown to significantly inhibit fibroblast proliferation either alone or in combination with β-agonists ^55, 56^. Similar anti-proliferative effects have also been reported with corticosteroids in ASM cells stimulated with distinct mitogenic agents ^57, 58^. However, others have shown that corticosteroids have no effect on ASM proliferation ^59, 60^. While corticosteroids inhibit growth factor–stimulated proliferation of ASM cells sourced from healthy controls, this effect was lacking on ASM cells from asthmatics ^59^. In animal models, dexamethasone has been shown to reduce goblet cell metaplasia; however, this treatment showed no effect on ASM mass and subepithelial fibrosis ^45^. In humans, in conjunction with limiting inflammation and airway hyperresponsiveness, corticosteroid treatment can also reduce mucin secretion and limit ECM deposition and AR ^61– 64^. However, others have shown that corticosteroids have a mixed effect on the resolution of subepithelial fibrosis ^65– 70^. Collectively, studies to date indicate a need for developing better therapeutic drugs for targeting AR pathology in obstructive lung diseases.

## New targets and approaches for airway remodeling

In recent years, basic science research has begun to provide insight into the mechanisms, mediated by multiple cell types, that promote AR and these studies help to inform potential strategies for managing AR. Certain approaches that show promise in mitigating features of AR have recently been proposed ( Table 3).

**Table 3.** Anti-remodeling effects of novel therapeutic approaches ( *in vitro*, animal, and human studies).

Class of therapeutic drugs	Target	Potential effect on airway remodeling (AR)
G protein–coupled receptor modulators	E-prostanoid receptors	Suppression of airway smooth muscle (ASM) proliferation ^72– 74^.
	Bitter taste receptors (TAS2Rs)	Regulation of ASM proliferation ^81, 82^. Reversal of allergen- induced AR features, including ASM mass ^83^. Alteration of mitochondrial function and induction of autophagy ^84^.
Biologics	Interleukin-5 (IL-5) cytokine	Reduced subepithelial fibrosis and extracellular matrix (ECM) deposition ^85, 86^.
	Immunoglobulin E	Reduced thickening of reticular lamina ^87^.
Mitogen-activated protein kinase (MAPK) inhibitors	MEK1 (MAPK kinase)	Regulation of mucus secretion ^88, 89^.
	p38	Reduced ASM mass and goblet cell metaplasia ^90^.
	c-Jun N-terminal kinases (JNKs)	Reduced mucus secretion and expansion of goblet cells ^91, 92^. Reduced proliferation of ASM and epithelial cells ^93^.
	Transforming growth factor- beta-activated kinase 1 (TAK1)	Reduced synthesis of IL-8 in ASM cells and reduced proliferation ^94, 95^.
Receptor tyrosine kinase inhibitors	Epidermal growth factor receptor	Reduced proliferation of ASM and epithelial cells ^96– 98^.
		Regulation of mucus secretion ^99– 102^.
		Reduced ASM thickening and goblet cell metaplasia ^103^.
	Platelet-derived growth factor receptor	Reduced ASM proliferation ^104^.
	Stem cell growth factor receptor (c-kit)	Attenuated collagen accumulation in lungs ^105^.
Non-receptor tyrosine kinase inhibitors	Spleen tyrosine kinase (Syk)	Reduced bronchial edema ^106^.
	Janus kinase (JAK)	Reduced expression of Gob-5 ^107^.
Other kinase inhibitors	TGF-β receptor type I (T-βRI) kinase	Diminished collagen deposition and reduced proliferation of ASM and epithelial cells in lungs ^108^.
	Rho-associated protein kinase (ROCK)	Curtailed ECM remodeling process ^109^.
Phosphodiesterase (PDE) inhibitors	PDEs	Marked reduction in subepithelial fibrosis and epithelial layer thickening ^110^. Reduced proliferation of ASM ^111^.

### (Other) G protein–coupled receptor ligands

G protein–coupled receptors (GPCRs) play a substantial role in numerous normal physiological functions. Unsurprisingly, they can contribute towards the pathophysiology of various diseases. As noted earlier, GPCR agonists (of the β2-adrenergic receptor) and antagonists (of the mAChRs and CysLTR) are principal drugs in the management of asthma and COPD. In this section, we provide a brief overview of novel targets, the drugs that modulate them, and the potential of such drugs to address AR pathology.

***E-prostanoid receptor agonists.*** The role of prostaglandin E _2_ (PGE _2_) and E-prostanoid (EP) receptor subtypes in mitigating AR has been a subject of recent research. Early studies demonstrated that autocrine PGE _2_, generated as a consequence of cytokine-induced cyclooxygenase-2 induction, significantly suppresses mitogen-induced ASM proliferation *in vitro* ^72^. Furthermore, studies of cultured human ASM in our laboratory have demonstrated that exogenous PGE _2_ shows relatively superior anti-mitogenic activity in comparison to multiple β-agonists with anti-mitogenic effects corresponding to drug efficacy in activating the cyclic adenosine monophosphate/protein kinase A (cAMP/PKA) axis ^73, 74^. Application of PGE _2_ in humans has been hindered by the ability of PGE _2_ to signal through multiple receptor subtypes (EP1–4) ^75– 79^, which contributes to undesirable side effects. It is now known that the cough response is mediated by PGE _2_ activation of the EP3 receptor on vagal sensory nerves ^80^. Additionally, studies in our lab have shown that EP3 receptor signaling has a pro-mitogenic role in ASM ^74^. To overcome the heterogeneity of PGE _2_ signaling, the development of EP receptor subtype–specific modulators that specifically promote Gs-cAMP-PKA axis activity (via EP2 and EP4 subtypes) has been purposed ^84, 112– 114^. Currently, our lab is evaluating various strategies of targeting specific EP receptor subtypes in pre-clinical models of allergen-induced asthma ^84^.

***Bitter taste receptors.*** Recently, our laboratory showed that bitter taste receptor (TAS2R) agonists can limit proliferation of ASM cells *in vitro* ^81, 82^. Mechanistically, TAS2R agonists restrict ASM proliferation by inhibiting (1) the growth factor–activated protein kinase B (Akt) phosphorylation; (2) transcription factors AP-1, STAT3, E2 factor, and NFAT; and (3) genes associated with cell cycle progression.

The anti-mitogenic effects of TAS2R agonists further translate to pre-clinical asthma models as well. In a chronic allergen (ovalbumin or house dust mite) challenge model, treatment with bitter taste compounds (chloroquine and quinine) significantly reversed remodeling features ^83^. Specifically, treatment with bitter compounds inhibited the expression of calponin, smooth muscle alpha-actin, and smooth muscle myosin heavy chain in lungs. Furthermore, levels of matrix metallopeptidase-8 (MMP-8) (neutrophil collagenase), pro-MMP-9 (gelatinase), and MMP-12 (macrophage metalloelastase) were significantly reduced in lungs following treatment with TAS2R agonists. Finally, allergen-induced expression of pro-fibrotic cytokine transforming growth factor-beta (TGF-β) as well as phospho-mothers against decapentaplegic homolog 2 (pSmad2) and fibronectin in the lung tissue was also curtailed by TAS2R agonists. Collectively, these studies indicate that TAS2R agonists, unlike current GPCR ligands used to treat asthma, address multiple features of asthma pathology, including AR. Advancements in the development of selective ligands for TAS2R subtypes will allow for a refined therapeutic approach in the near future.

Bitter tastants have also been shown to modulate function of ciliated epithelial cells ^115^. Specifically, the motile cilia on human airway epithelia express TAS2Rs (T2R4, T2R43, T2R38, and T2R46). The organization of TAS2Rs on cilia with the distribution of the signaling machinery along the ciliary shaft and within the attached epithelial cell presents an interesting mechanical apparatus for signal transduction. Stimulation of TAS2Rs with bitter tastants induces transient Ca ^2+^ flux within the epithelial cells and increases ciliary beat frequency. Functionally, promoting increased ciliary movement could be beneficial in the removal of excess mucus from the airways.

In recent years, the role of mitochondrial dysfunction in disease states, including obstructive lung diseases, has become increasingly clear ^116, 117^. Specifically, a role for autophagy/mitophagy in regulating mitochondrial function in pathophysiology of obstructive lung diseases is emerging. As noted earlier, our studies show that activation of TAS2Rs can promote anti-mitogenic activities. Further explorations into the mechanisms that underlie the anti-mitogenic effects of TAS2R agonists have uncovered an interesting role for bitter tastants in altering mitochondrial function and inducing autophagy ^82^. TAS2R agonists can induce changes in mitochondrial membrane potential, increase reactive oxygen species generation, and promote mitochondrial fragmentation. These observations provide insight into the broader therapeutic potential of targeting mitochondrial function and promoting autophagy to restrict cellular proliferation.

### Biologics

Asthma pathology is orchestrated by multiple immunologic mediators (cellular and secreted) ^118^. Consequently, in recent years, therapies that target specific cytokines or immune cells to disrupt immune networks responsible for asthma pathology have gained significant interest. Targeting key cytokines with specific antibodies—biologics, including antibodies targeting interleukin-4 (IL-4), IL-5, and IL-13—can significantly limit recruitment of inflammatory cells to the lungs or blunt their pleiotropic effects. For instance, anti–IL-5 treatment can significantly reduce the number of circulating eosinophils in asthmatics and improve lung function ^119– 121^. Anti–IL-5 treatment has been shown to prevent the development of subepithelial fibrosis in a murine model of asthma and reduce incorporation of proteoglycans in the human airway wall ^85, 86^. However, antibodies targeting other cytokines or their receptors have not been studied in the context of AR ^122^. Collectively, the data on the effects of biologics on AR are lacking and this is possibly due to the relatively recent development of these drugs. Future longitudinal studies that evaluate biologics in the context of remediation of AR features are needed to address the utility of these drugs as anti-AR agents.

Allergen-specific immunoglobulin E (IgE) isotype antibodies can cross-link on mast cells and basophils, causing degranulation and release of histamine, cytokines, and growth factors ^123^. Biologics that block the interactions of IgE antibodies to the high-affinity FcεRI receptors on mast cells and antigen presenting cells can curtail the sensitization profile in asthmatics and reduce exacerbations. In severe asthmatics evaluated for 36 months, blocking IgE activity was sufficient to reduce the thickening of the reticular lamina, thereby having an impact on AR ^87^.

Although biologics have become an increasingly important tool in the management of severe asthma, their application in the clinic has some drawbacks ^124– 126^. Application of cytokine-specific antibody therapy is limited by the heterogeneity of asthma phenotypes ^127^. Non-atopic asthmatics are also not suitable for certain therapy (for example, anti-IgE). Biologics can also cause side effects such as hypersensitivity reactions, although the underlying mechanisms are unclear. Finally, there is also significant cost associated with the use of biologics.

### Kinase inhibitors

Kinase enzymes modulate multiple cellular functions by regulating various signaling networks, including those regulating cellular proliferation and growth. Consequently, inhibitors that target various kinases have received increasing attention. Modulators of kinase functions account for one third of drugs in the development pipeline, and the majority of these represent cancer therapeutics ^128^. In this section, we provide a brief overview of drugs that target distinct kinases. For a more comprehensive discussion of targeting kinases in the context of obstructive lung diseases, the reader is referred to ^129^.

***Mitogen-activated protein kinase inhibitors.*** Mitogen-activated protein kinases (MAPKs) have been studied extensively for their contribution to inflammatory gene expression and activation of multiple networks that contribute to the pathophysiology of obstructive lung diseases ^130^. Extracellular signal-regulated kinases (ERK1/2) are particularly interesting given that they are activated in multiple cell types that contribute to asthma and COPD pathology ^88, 131, 132^. Inhibition of ERK kinase (MAPK1, or MEK1) which is upstream of ERK1/2 can significantly reduce mucin 5AC, oligomeric mucus/gel-forming ( *MUC5AC*) expression in cultured human bronchial epithelial cells subjected to chronic mechanical stress at the air-liquid interface ^88, 89^. Other MAPKs, such as p38, c-Jun N-terminal kinase (JNK), and transforming growth factor beta-activated kinase 1 (TAK1), are activated in asthma and COPD ^129^, and inhibitors of these targets can mitigate various features of AR in both cell and animal asthma models ^90– 95^. However, to date, no studies in humans have evaluated these inhibitors. Another limitation of kinase inhibitors is that these compounds are predominantly inhibitors of ATP-competitive and catalytic sites and block all enzymatic activity, including MAPK functions important for normal physiological activity in cells. Given the ubiquitous functions of the MAPK signaling pathway, development of substrate-selective MAPK inhibitors with the goal of targeting specific kinase functions associated with disease, while preserving kinase functions in normal cells, appears necessary to overcome limitations of off-target effects.

***Receptor tyrosine kinase inhibitors.*** Receptor tyrosine kinases (RTKs) occupy a central role in critical signaling networks that promote asthma pathology, including remodeling ^133^. With inflammation, distinct RTKs and their ligands (for example, epidermal growth factor) are upregulated in human asthmatic airways and show a strong correlation with disease severity ^134– 143^. RTKs can stimulate pathophysiological functions in ASM and epithelial cells. Thus, significant interest in advancing tyrosine kinase inhibitors for targeting RTKs has developed.

Activation of epidermal growth factor receptor (EGFR) is essential for mucus secretion and goblet cell metaplasia ^144^. It is also responsible for sustaining oxidative damage in the epithelial compartment through recruitment of neutrophils in a TGF-β–dependent manner ^145– 148^. EGFR inhibitors tyrphostin AG1478 and BIBX1522 have been evaluated *in vitro* and in animal models of lung inflammation ^99– 101^. Collectively, these studies report significant reductions in expression of mucus-associated *MUC5AC* gene and mucin secretion. More importantly, there is a concomitant reduction in collagen deposition and ASM proliferation ^96– 98^. Although these observations are encouraging, some inhibitors of EGFR have failed to produce similar outcomes in clinical studies ^149^. Activation of platelet-derived growth factor receptor (PDGFR) has been shown to stimulate ASM proliferation *in vitro* and *in vivo* ^138, 150, 151^. Multiple drugs targeting PDGFR can mitigate ASM proliferation *in vitro*, although animal and human studies that address AR are lacking ^104^. In severe corticosteroid-dependent asthmatics, treatment with the tyrosine kinase inhibitor mastinib has shown improved outcomes; however, some adverse effects, including skin rash and edema, have also been reported ^152^. During airway inflammation, multiple cell types (immune and resident) can be stimulated to secrete angiogenic factors, including vascular endothelial growth factor (VEGF) ^153– 159^. These angiogenic factors can further stimulate increase in formation of new blood vessels from endothelial cells in the subepithelial mucosa ^160– 162^. Although the contribution of neovascularization in AR is unclear, it has been suggested that newly formed vasculature is permeable, thus contributing to edema and limiting airflow ^71, 161, 163, 164^. An antagonist of VEGFR-1 and VEGFR-2 (SU5416) has been shown to limit inflammatory responses in animals ^165^, although its impact on AR is unknown and studies in humans are lacking.

Because multiple RTKs contribute to pathology of AR, a novel strategy that targets multiple RTKs has gained momentum. In a pre-clinical murine model of ovalbumin-induced asthma, treatment with nintedanib—a small-molecule inhibitor that targets multiple RTKs (VEGFR, fibroblast growth factor receptor, and PDGFR)—significantly improved indices of remodeling and airway inflammation ^166^. This approach could be useful in targeting multiple redundant RTK networks that contribute to AR. Finally, inhibitors of non-RTKs, such as stem cell growth factor receptor (c-kit), spleen tyrosine kinase (SYK), the proto-oncogene tyrosine-protein kinase Src, and Janus kinase (JAK), have also been investigated in rodent models of asthma but have not yet progressed to studies in humans ^105, 106, 167– 171^.

***Other kinase inhibitors.*** Diverse stimuli (cytokines, viruses, growth factors, free radicals, and so on) can activate the transcription factor nuclear factor-kappa B (NF-κB) in multiple airway cell types. This transcription factor plays a key role in orchestrating immune responses and thus multiple intra- and inter-cell inflammatory signals ^129^. Although inhibitors that target activation of NF-κB have been shown to suppress certain synthetic functions of ASM ^172^ and modulate pro-inflammatory outcomes in epithelial cells ^173^, specific NF-κB inhibitors have not translated into clinical trials for asthma and this is due to their multiple side effects ^129^. Inhibitors of phosphatidylinositol-4,5-bisphosphate 3-kinase (PI-3K) that regulate cellular lipids and coordinate inflammatory pathways have undergone extensive investigation in asthma and COPD ^174, 175^. However, data assessing AR indices are lacking. TGF-β plays an important role in cellular proliferation and differentiation and its expression increases in asthmatic airways, especially in the submucosal compartment ^176– 178^. TGF-β has also been implicated in AR and can promote proliferation in ASM ^179, 180^. TGF-β activates TGF-β receptor type I (T-βRI) kinase, which in turn activates Smad-dependent signaling that regulates expression of various genes. Small-molecule inhibitors of (T-βRI) kinase have yielded mixed results in studies assessing their effects on mechanisms mediating AR. T-βRI kinase inhibitors have been shown to diminish collagen deposition in lungs of rats challenged repeatedly with an allergen ^108^. *In vitro*, T-βRI inhibitors have demonstrated the ability to limit ASM proliferation, although their use in animal studies failed to inhibit TGF-β–induced increases in ASM mass ^108, 181^. Clinical application of these inhibitors has been limited due to adverse effects, including cardiotoxicity in clinical trials for cancer therapy ^182^. Protein kinase C (PKC) is another target of interest given its ability to promote contractile signaling in ASM following activation of Gq-coupled GPCRs. PKC is also relevant to AR because of its role in ASM proliferation ^183^ and mucus secretion in epithelium ^148^. Owing to severe toxicity, non-selective inhibitors of PKC have not progressed to clinical trials ^129, 184^. Selective inhibitors of PKC isoforms have been developed for clinical studies for treatment of cancer, metabolic diseases, and psychiatric disorders, although adverse effects have been problematic and no clinical trials have been conducted for treatment of obstructive lung diseases in humans ^129, 184^. Finally, Rho-associated protein kinase (ROCK) inhibitors have been shown to mitigate multiple features of asthma, including AR in guinea pig and murine models ^109, 185^. Although ROCK inhibitors have been approved for certain indications, clinical trials in asthma or COPD are lacking ^129^ as issues regarding selectivity and toxicity have limited progression of these inhibitors to clinical application in airway diseases ^129^.

In summary, *in vitro* studies of pharmacological inhibition of multiple kinases that contribute to dysfunction in ASM and epithelium have yielded promising results ^181, 186^. However, certain limitations have stalled progression of many drugs for clinical use. Specificity, efficacy, solubility issues, and poor pharmacokinetic profiles plague drug development ^187^. With chronic inhibitor treatment, compensatory signaling by other kinases may limit drug efficacy; this appears to be the case with p38 isoform inhibitors ^129, 188^. Inhibition of any widely expressed kinase runs the risk of adverse effects. For example, given that NF-κB is crucial for mounting an immune response to microbial pathogens, blocking its activation could render patients susceptible to life-threatening infections ^129^. Current challenges in developing effective and safe kinase inhibitors hinge on improving the poor solubility, selectivity, and targeting of the current versions of these drugs ^129^.

### Other small-molecule inhibitors

Phosphodiesterase (PDE) inhibitors have a beneficial effect of promoting ASM relaxation by increasing intracellular cAMP resulting in PKA-mediated ASM relaxation. In murine models of asthma, PDE inhibitors have also been shown to curtail inflammation and reduce AR ^110^. Inhibition of PDE3 (but not PDE4) has anti-proliferative action on mitogen-activated human ASM cells *in vitro*, but as with most potential anti-AR drugs, useful *in vivo* data are lacking ^111^. More recently, an inhibitor of PDE8 (PF-04957325) has been shown to regulate proliferation of ASM cells by enhancing cAMP accumulation generated specifically from the β2-AR/AC6 pathway ^189^.

The rapamycin derivative SAR-943 has been shown to limit the mitogen-induced proliferation of human ASM cells (but not human epithelial cells) *in vitro* and mitigate inflammation and AR *in vivo* in ovalbumin-challenged mice ^190^, yet no clinical studies for asthma or COPD have been reported.

## Conclusions

The correlation between AR and obstructive lung disease severity suggests a strong pathogenic role of AR in these diseases. Thus, remediation of AR appears critical for improving the severity and progression of these diseases. A growing arsenal of small-molecule inhibitors and biologics in conjunction with non-pharmacological interventions such as bronchial thermoplasty has shown promise in addressing this unmet clinical need. As our understanding of mechanisms underlying AR improves, so will the drug development approaches as well as the phenotyping capabilities that accurately assess AR in humans. These advances will undoubtedly fulfill our need for more refined, efficacious, and safer drugs that enable us to finally control the entire spectrum of asthma pathology.

## Abbreviations

AR, airway remodeling; β _2_-AR, beta 2 adrenergic receptor; ASM, airway smooth muscle; cAMP, cyclic adenosine monophosphate; c-kit, Stem cell growth factor receptor; COPD, chronic obstructive pulmonary disease; CysLTR, cysteinyl leukotriene receptor; ECM, extracellular matrix; EGFR, epidermal growth factor receptor; EP, E prostanoid; ERK1/2, extracellular signal-regulated kinases 1/2; GPCR, G protein–coupled receptor; FGFR, fibroblast growth factor receptor; ICS, inhaled corticosteroids; IgE, immunoglobulin E; IL, interleukin; JAK, Janus kinase; JNK, c-Jun N-terminal kinase; mAChR, muscarinic acetylcholine receptor; MAPK, mitogen-activated protein kinase; MEK1, mitogen-activated protein kinase kinase 1; MMP, matrix metalloproteinase; MUC5AC: mucin 5AC, oligomeric mucus/gel-forming; NF-κB, nuclear factor-kappa B; PDE, phosphodiesterase; PDGFR, platelet-derived growth factor receptor; PGE _2_, prostaglandin E _2_; PI-3K, phosphatidylinositol-4,5 bisphosphate 3-kinase; PKA, protein kinase A; PKC, protein kinase C; ROCK, Rho-associated proteinase kinase; RTK, receptor tyrosine kinase; Smad2: Mothers against decapentaplegic homolog 2; Syk, spleen tyrosine kinase; TAK1, Transforming growth factor-β-activated kinase 1; TAS2R, bitter taste receptor; TGF-β, transforming growth factor-beta; T-βRI, transforming growth factor-beta receptor type I kinase; VEGFR; vascular endothelial growth factor receptor
